# Effectiveness and cost-effectiveness of a guided Internet- and mobile-based intervention for the indicated prevention of major depression in patients with chronic back pain—study protocol of the PROD-BP multicenter pragmatic RCT

**DOI:** 10.1186/s12888-017-1193-6

**Published:** 2017-01-21

**Authors:** L. Sander, S. Paganini, J. Lin, S. Schlicker, D. D. Ebert, C. Buntrock, H. Baumeister

**Affiliations:** 1 Institute of Psychology, Department of Rehabilitation Psychology and Psychotherapy, University of Freiburg, Engelbergerstr. 41, D-79085 Freiburg, Germany; 2 Medical Faculty, Medical Psychology and Medical Sociology, University of Freiburg, Hebelstraße 29, Freiburg, 79104 Germany; 3 Institute of Psychology, Department of Clinical Psychology and Psychotherapy, University of Erlangen-Nürnberg, Nägelsbachstr. 25a, D- 91052 Erlangen, Germany; 4 Institute of Psychology and Education, Department of Clinical Psychology and Psychotherapy, University of Ulm, Albert-Einstein-Allee 47, D-89069 Ulm, Germany

**Keywords:** Prevention, RCT, eHealth, Internet and mobile based, Major depression, Chronic back pain, CBT, Effectiveness, Economic evaluation

## Abstract

**Background:**

Reducing the disease burden of major depressive disorder (MDD) is of major public health relevance. The prevention of depression is regarded as one possible approach to reach this goal. People with multiple risk factors for MDD such as chronic back pain and subthreshold depressive symptoms may benefit most from preventive measures. The Internet as intervention setting allows for scaling up preventive interventions on a public mental health level.

**Methods:**

This study is a multicenter pragmatic randomized controlled trial (RCT) of parallel design aiming to investigate the (cost-) effectiveness of an Internet- and mobile-based intervention (IMI) for the prevention of depression in chronic back pain patients (PROD-BP) with subthreshold depressive symptoms. eSano BackCare-DP is a guided, chronic back pain-specific depression prevention intervention based on cognitive behavioral therapy (CBT) principles comprising six weekly plus three optional modules and two booster sessions after completion of the intervention. Trained psychologists provide guidance by sending feedback messages after each module. A total of 406 patients with chronic back pain and without a depressive disorder at baseline will be recruited following orthopedic rehabilitation care and allocated to either intervention or treatment-as-usual (TAU). Primary patient-relevant endpoint of the trial is the time to onset of MDD measured by the telephone-administered Structured Clinical Interview for DSM (SCID) at baseline and 1-year post-randomization. Key secondary outcomes are health-related quality of life, depression severity, pain intensity, pain-related disability, ability to work, intervention satisfaction and adherence as well as side effects of the intervention. Online assessments take place at baseline and 9 weeks as well as 6 and 12 months post-randomization. Cox regression survival analysis will be conducted to estimate hazard ratio at 12-month follow-up. Moreover, an economic analysis will be conducted from a societal and public health perspective.

**Discussion:**

This is the first study examining an IMI for depression prevention in a sample of chronic pain patients. If this implementation of a depression prevention IMI into orthopedic aftercare proves effective, the intervention could be integrated into routine care with minimal costs and extended for use with other chronic diseases. Results will have implications for researchers, health care providers and public health policy makers.

**Trial registration:**

The trial is registered at the WHO International Clinical Trials Registry Platform via the German Clinical Studies Trial Register (DRKS): DRKS00007960. Registered 12 August 2015.

## Background

Major depressive disorder (MDD) is related to high disease burden for both people affected and society [1]. In a recent literature review investigating global variation in the prevalence and incidence of MDD, a global point prevalence of 4.7%, a lifetime prevalence between 10 and 15% and a global incidence of 3.0% have been reported [2, 3]. It is estimated that existing psychological and pharmacological treatments have the potential to avert only 36% of the burden of MDD, and only when assuming perfectly efficient provision of existing treatments in terms of coverage, patient compliance, an d clinician competence [4, 5]. Thus, we are either in need of more powerful interventions for treating depression or we should aim at diminishing the likelihood of developing depression in the first place, highlighting prevention of depression as a promising approach. Recent research suggests that psychological preventive interventions such as cognitive behavioral therapy (CBT) or interpersonal psychotherapy have the potential to prevent a clinically significant number of new depression cases [6]. A meta-analysis of 32 randomized controlled trials (RCTs) reported a reduced incidence rate for MDD of 21% (incidence rate ratio = 0.79, 95% confidence interval: 0.69–0.91) when comparing psychotherapy-based preventive interventions with usual care or wait list conditions.

While the effectiveness of preventive interventions seems sufficiently documented, it remains challenging to identify target populations that benefit most from preventive measures [7]. According to Cuijpers and colleagues [8] two factors need be taken into account: the “impact” and the “effort” of preventive measures. An adequate “impact” means that prevention must lead to a substantial reduction of total disease burden. Therefore, a substantial proportion of new cases must be prevented if assembled risk indicators are fully blocked. A reasonable level of “effort” is primarily defined as a low number needed to be treated (NNT) to prevent one new case of MDD. Additionally, persons at risk should be easily identifiable and interventions should not only be cost-effective but also low priced to allow for their implementation at a population level.

From this viewpoint, chronically medically ill patients appear to be a meaningful target population for the prevention of MDD, given the substantially increased prevalence for MDD in this population compared to the general population [9, 10]. In addition, comorbid MDD in medically ill patients is associated with numerous negative implications such as problems in the physician-patient relationship, increased risky health-related behaviors, higher medical symptom burden, medical complications, lowered quality of life and increased mortality [11, 12]. Within the group of medically ill persons, back pain is one of the most common conditions [10, 13] and is associated with a two to three-fold increased risk for MDD [14]. In addition, depression is one of the core predictors of persistent pain symptoms, increased pain related disability, and poor treatment outcomes, and is associated with increased morbidity and health care costs as well as diminished quality of life [11, 12, 15–17].

The benefits of prevention can be multiplied by focusing on patients who already show some depressive symptoms due to several reasons. First, subthreshold depressive symptoms are an additional risk indicator for MDD [18, 19]. Multiple risk groups have increased specificity for prevention measures which leads to a reduction of NNT (“effort”) [20] and leading to greater cost-effectiveness of preventive interventions. Second, by lowering the NNT, the number of persons who are not in need of a preventive intervention, but receive it, will be reduced. Third, subthreshold depression itself is a considerable disease burden for people affected and for society [21, 22]. A successful preventive intervention will not only reduce the risk of developing MDD but also improve depression symptom severity at all levels of depression, as shown by a recent meta-analysis (pooled effect size g = 0.35, 95%-CI: 0.23–0.47; [23]). Fourth, uptake rates of a depression prevention intervention may be higher in a target population of patients with depressive symptoms, as treatment utilization was found to be associated with severity of baseline depression [24].

The internet is an appropriate prevention medium for scaling up preventive interventions as units of delivery are reasonably priced and can be easily administered [7, 25]. It has several additional advantages as discussed elsewhere [26–28].

In prior studies, Internet- and mobile-based interventions (IMI) have shown to be effective in the treatment of MDD [29, 30] as well as in the treatment of subthreshold depression, indicating their potential to be utilized for preventive interventions [25, 31–34]. Human support (guidance) has repeatedly been shown to have a positive effect on effectiveness of and adherence to IMIs [35, 36].

The proposed study aims to investigate the effectiveness and cost-effectiveness of an IMI (eSano BackCare-DP) to prevent the onset of depression for chronic back pain patients (PROD-BP) with subthreshold depressive symptoms. The study will be embedded into routine orthopedic care in order to examine the intervention’s effectiveness in an unselected sample of all eligible chronic back pain patients (i.e. the implementation will not be limited to a self-selected population of people who are already attracted to depression prevention interventions and the Internet). It is expected that

1. eSano BackCare-DP is effective in preventing the onset of MDD compared to treatment as usual (TAU) over a 12-month follow-up period,
2. eSano BackCare-DP is cost-effective compared to TAU.
3. Compared to TAU, eSano BackCare-DP is superior in terms of (a) depression response, (b) work capacity, (c) quality of life, (d) pain related disability and (e) pain intensity.

Furthermore, the distributions of principal confounders in each group will be explored.

## Methods

### Study design

**Fig. 1:**
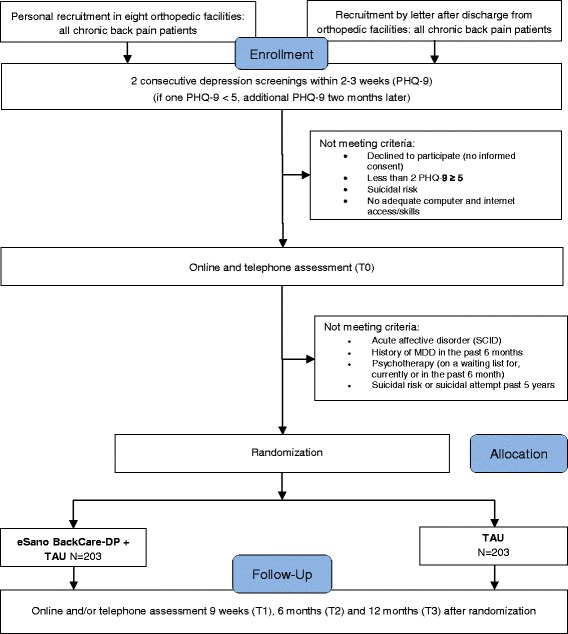
Flow chart of inclusion and study procedure

This project is a multicenter randomized controlled clinical trial (RCT) of parallel design comparing the effectiveness and cost-effectiveness of a guided depression prevention IMI with treatment as usual (TAU) (Fig. 1). All participants will receive TAU. Participants of the intervention group will additionally receive the IMI eSano BackCare-DP.

This clinical trial will be conducted and reported in accordance with the CONSORT-supplement for pragmatic RCTs [37] and the guidelines for executing and reporting internet-based intervention research [38]. In order to guarantee data quality and safety, the Clinical Trials Unit Freiburg will perform monitoring visits to the recruitment centres before, during and after completion of the study. Moreover, an independent Data Safety and Monitoring Board (DSMB) has been established. It consists of two experienced scientists and psychotherapists (MHä, MHa) and a statistician (LK) with long-standing experience in clinical trials.

### Inclusion and exclusion criteria

Patients who provide written consent will be included in the study if they meet the following criteria: a) age 18 and above, b) presence of chronic back pain assessed by physician diagnosis and participants report on pain chronicity (>6 months), c) sufficient knowledge of German language, d) internet access, e) persistent subthreshold depressive symptomatology (Patient Health Questionnaire (PHQ-9) ≥ 5 in two consecutive screenings within 2–3 weeks. If only one PHQ is over cut-off, a third PHQ will be administered 2 months later.

Patients will be excluded who meet DSM criteria for a) current depressive episode or depressive episode within the last 6 months (following Kupfer [39]), b) current dysthymia, c) current or lifetime bipolar disorder. Additionally patients will be excluded in case of d) participation in ongoing psychotherapy, completed psychotherapy in the past 6 months, or being on a waiting list for psychotherapy (beginning within 3 months), e) currently suicidal or reporting suicidal attempts within the past 5 years. Patients with a diagnosis of any affective disorder will receive information on possible mental health care options, including the offer to take part in a parallel clinical trial for the treatment of depression in patients with chronic back pain and clinical depression [40]. In cases of severe depression, a trained psychotherapist from the study team [HB, SaS, LS] will contact the participant to initiate further actions.

### Setting/Recruitment

Recruitment has started in October 2015. Recruitment will continue until the target sample size has been reached. Participants are recruited in the aftermaths of their orthopedic rehabilitation care. In order to increase the representativeness of our sample, we established two comparable recruitment strategies. First, back pain patients from eight orthopedic rehabilitation units are screened at admission and discharge. Clinical staff informs and recommends participation to patients screened positive (personal recruitment). In addition, an information flyer and a patient information form with a detailed description of the study process and information on the intervention are provided. Patients providing their informed consent are contacted by the study team in order to clarify further eligibility criteria by means of an online- and telephone assessment including a telephone administered clinical interview (SCID; [41–43]).

Second, back pain patients from orthopedic rehabilitation units across Germany receive a letter with the same information flyer and study process information (recruitment by letter). Interested back pain patients fill out an online PHQ-9 screening. Positive screened patients (PHQ-9 ≥ 5) providing their informed consent conduct the aforementioned online- and telephone assessment including a second PHQ-9 screening to ascertain persistent depression in line with the first recruitment strategy (second PHQ-9 ≥ 5). A third PHQ-9 screening takes place 2 months afterwards in case of only one PHQ-9 being screening positive. Eligible back pain patients from both recruitment strategies receive an email providing further information and a link referring to the intervention website. All participants are free to seek any additional help during the trial. Trial participants receive 15€ for the completed follow-up telephone assessment.

### Randomization

Participants eligible for inclusion will be randomly allocated to one of two groups (intervention or TAU). Randomization and allocation will be prepared in advance by a researcher (SaS) who is responsible for administration of the trial and participants. This researcher will remain blinded to all processes within the intervention. An automated, web-based randomization program (www.sealedenvelope.com) will be used, which features permuted block randomization, variable block sizes of 4,6,8 (randomly arranged), and an allocation ration of 1:1.

## Intervention

### Intervention condition

**Fig. 2:**
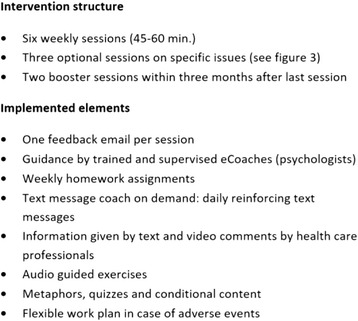
Intervention structure, technical implementations and support

**Fig. 3:**
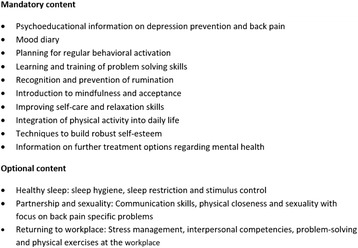
Intervention content, based on Cognitive Behavioral Therapy (CBT), including back pain specific self-management

The intervention (eSano BackCare-DP) consists of six weekly sessions plus three optional modules and two booster sessions of approximately 45 to 60 min each (see Figs. 2 & 3). Participants can decide when to complete the booster sessions at the end of the last session. They can choose two dates within a timeframe of 3 months following the intervention, and will be reminded to log-in again. Sessions can be repeated as often as desired. All modules consist of information about depression and (chronic) back pain, provided by text, audio, and video, as well as assignments, metaphors and exercises. The content of the intervention is based on cognitive behavioral therapy (CBT) for depression, including elements of psychoeducation, social skills, problem solving, behavioral activation, improving self-care, relaxation and motivation for physical exercises. The intervention is based on prior interventions that have been evaluated in a number of RCTs in different samples [31, 34, 44–46]. We adapted and substantially extended the predecessors to the context of depression prevention and chronic back pain. In order to address chronic back pain patients specifically, psychological pain intervention elements are integrated in every module of the intervention. Moreover, three optional modules are offered, focusing on problems with sleep, partnership/sexuality and returning to the workplace. Emphasis is laid on homework assignments, which are intended to provide practice of learned skills. To enhance patient adherence, interactive elements (quizzes, conditional contents) are implemented. Participants will be asked about adverse events at the beginning of every session with the advice to check whether it is the right time to go on with the session. The intervention platform is provided by Minddistrict (www.minddistrict.com), a company specialized in the provision of web-based health interventions. Access to the platform proceeds through a unique username-password combination and will be available on a 24/7 basis. All transferred data will be secured based on ISO27001 and guidelines NEN7510.

### Text message coach

At the beginning of the training, participants are asked if they want to receive daily reinforcing text messages during the 6-week training period. Text message prompts have been shown to be beneficial in internet interventions with positive effects on efficacy and adherence [47–49]. The messages are sent automatically and coordinated with intervention content in order to integrate the learned techniques into daily life of the participants. Message content aims at reminding patients to complete homework assignments, repeating training content, and reinforcing motivation of participants.

### Guidance

Trained and supervised (by HB, JL, SP) psychologists (eCoaches) will guide participants during the training by providing a semi-standardized feedback within 2 working days after each completed session, using an eCoach manual. The eCoach manual is standardized to ensure protocol adherence by the eCoaches. All communication between eCoaches and participants runs via the intervention platform. Guidance time spent per participant per intervention will be measured to provide data for the economic analyses of the project. The feedback content will match the participants’ assignments and provide support for treatment adherence. Feedback also includes positive reinforcement to encourage participants to continue with the training. If any further questions arise, participants and eCoaches can contact each other at any time via the intervention platform. eCoaches will also send reminders to participants, who do not complete intervention modules on time.

### Control condition

Participants of the control condition will have unrestricted access to TAU. Although national treatment guidelines for low back pain and depression exist [50], TAU following orthopedic rehabilitation care may vary. There is no minimum treatment defined and TAU will not follow a standardized protocol; however, all received types of medical/psychological help during the last 3 months will be monitored with the Trimbos/iMTA questionnaire, to account for all costs associated with psychiatric illness (TIC-P) [51]. Using these data, an accurate description of TAU can be provided.

### Sample size/power calculation

The aim of the study is to compare the effectiveness of the intervention against TAU using a cox regression survival analysis with a significance level of 5%. Based on previous studies of depression incidence in chronic back pain patients and subclinical depression, we expect a mean incidence of MDD of 20% in the control group within the 12-months follow-up period [52–55]. Based on a prior conducted trial, we assume an absolute risk reduction of 9% with a respective hazard ratio of 0.522 between treatment and control group at 12 months after randomization [34]. A total of 64 events (event = onset of depression) need to be observed to detect a 47.8% decrease in hazard (hratio = 0.522) of the treatment group relative to the hazard of the control group based on a power of 80%. Accordingly, 406 participants (203 per group) need to be randomized (calculated using Stata/SE 13.1). The inclusion of relevant baseline predictors of depression onset will further increase power (e.g. baseline depression severity). Recruitment will be continued to allow for an expected attrition rate of 20%.

## Assessments

Assessments will be conducted at pre-treatment (T0) and at 9 weeks, 6- and 12-months follow-up (T1, T2, T3; see Table 1). All self-report assessments, potentially effect-modifying covariates and demographic variables will be provided using a web-based interface integrated into the intervention platform. Section A (Affective Syndromes) of the SCID [43], the Hamilton Rating Scale for Depression (HAM-D-17 [56]) and the Quick Inventory of Depressive Symptomatology (QIDS-C16; [57]) will be performed via telephone interviews at T0 and T3. Moreover, we translated the SCID-V-RV into German to be able to comment on DSM-IV and -V [58] diagnoses.

**Table 1:** Outcome assessments and assessment time point

Variables	Measurement	Screening	T0	T1	T2	T3
Inclusion/exclusion criteria						
Chronic Back pain	MR + TI	x	x			
Depressive symptomatology	PHQ-9	x	x	x	x	x
Inclusion criteria a), c), d)	TI/SRQ		x			
Acute/past 6 months depressive episode, dysthymia or bipolar disorder	SCID		x			x
Current/past 6 months/on waiting list for psychotherapy	TI		x			
Suicidality	SCID/HAM-D/QIDS		x			x
	PHQ-9	x	x	x	x	x
Primary outcome						
Onset of Depression	SCID		x			x
Secondary outcomes						
Severity of depressive symptoms	PHQ-9	x	x	x	x	x
	HAM-D/QIDS		x			x
Quality of life	AQOL-6D/EQ-5D-5 L		x	x	x	x
Pain intensity	Rating scale		x	x	x	x
Pain related disability	ODI		x	x	x	x
Pain self-efficacy	PSEQ		x	x	x	x
Ability to work	SPE		x	x	x	x
Economic evaluation						
Costs	TiC-P		x		x	x
Quality of life	AQOL-6D/EQ-5D-5 L		x	x	x	x
Covariates						
Demographic variables	SRQ/MR	x	x			
Depression type and chronicity	SCID		x			x
Patient adherence	Attrition rate			x	x	
Patient satisfaction	CSQ-8^a^			x		
Side-effects of intervention	INEP^a^			x	x	
Back Pain type and chronicity	MR	x				
Internet affinity	IAS		x			

*AQol*-*6D* Assessment of Quality of Life, *CSQ*-*8* Client Satisfaction Questionnaire, *EQ*-*5D*-*5 L* European Quality of Life scale, *HAM*-*D* Hamilton Rating Scale for Depression, *IAS* Internet Affinity Scale, *INEP* Inventory for the Assessment of Negative Effects of Psychotherapy, *MR* Medical record, *ODI* Oswestry Disability Index, *PHQ*-*9* Patient Health Questionnaire, *PSEQ* Pain Self-Efficacy Questionnaire, *SCID* Structured Clinical Interview for DSM, *SPE* Subjective Prognostic Employment scale, *SRQ* Self-report assessment questionnaire, *TI* telephone interview, *TiC*-*P* Trimbos/iMTA questionnaire for costs associated with psychiatric illness

^a^intervention group only

Videos promoting the importance of collecting data will be implemented into online assessments to enhance compliance with completing measures. In psychological intervention trials, blinding of study participants and eCoaches is not possible. However, all members of the research team conducting telephone administered outcomes will remain blinded. Therefore, both participants and interviewers will be reminded of the reason and importance of blinding at the beginning of each interview. Moreover, performance bias will be minimized, as the web-based intervention is separated from other health care services.

### Procedure on suicidal ideation

The telephone interviews (SCID, HAM-D, QIDS) and questionnaires (PHQ-9) include a suicide screening to identify participants who are currently suffering from suicidal ideation. We will follow a suicide protocol adapted from prior trials [31, 44] if participants score on any suicidality item. Participants who report low suicidal ideation (HAM-D, QIDS or PHQ-9 item score = 1) will receive an email with detailed information on available health services and the advice to seek professional help if symptoms increase. If participants express moderate to high suicidal ideation during the assessment or express any suicidal thoughts or intentions to their eCoach, a trained psychotherapist from the study team [EM, LS, HB, SaS] will contact the participant and initiate further actions.

## Outcome measurements

### Primary outcome: Time to onset of MDD

To assess the time to onset of MDD within the 12-month follow-up period, the depression related modules of SCID will be part of the telephone assessment [41, 59]. The SCID is a comprehensive, structured interview designed to be used by trained interviewers for the determination of mental disorder diagnoses according to the definitions and criteria of DSM. It enables a reliable, valid and efficient assessment of depressive disorders [59]. Inter-rater reliability of the SCID was reported to be moderate to high and high for inter-rater reliability comparing telephone and face-to-face interviews [60, 61].

Interviewers are trained and weekly supervised by clinical psychologists (LS, EM) and are blinded to randomization condition. After the training period, supervisors and the interviewers assess participants together, with comparison of results as follows: The Inter-Rater Reliability (IRR) for the SCID, measured by *Cohen’s kappa* and the *Intra-Class Correlation* for the HAM-D and QIDS. An almost perfect Cohen’s kappa ≥ .81 [62] and an excellent ICC coefficient ≥ .75 [63] are considered as sufficient. Moreover, the interviewers are compared to each other on a random basis to assess the IRR.

Time to onset of MDD will be assessed using life charts. Therefore, life events will be recalled using a calendar method to determine presence of symptoms at each month within the follow-up period. Supervisors are blinded to participants’ group allocation.

### Secondary outcomes

#### Depressive symptoms

Depressive symptoms will be assessed by the self-administered Patient Health Questionnaire (PHQ-9) [64], a telephone-based clinician rating of the Hamilton Rating Scale for Depression (HAM-D-17), and the Quick Inventory of Depressive Symptomatology (QIDS) [56, 57]. The PHQ-9 is a well validated and widely used depression screening instrument [64] and has also been evaluated to be delivered as online-version [65]. The 17-item HAM-D is the most widely used clinician-rated measure of depression severity and as such viewed as the gold standard for the assessment of depression severity. The 16-Item QIDS will be used to further validate the depressive symptom outcome measures. It covers all criterion symptom domains of the DSM for diagnosing a MDD [57]. HAM-D and QIDS are administered to determine depression response [66].

#### Quality of life

To assess health-related quality of life, the Assessment of Quality of Life (AQoL-6D) will be used, which includes 20 items assessing the following dimensions: independent living, mental health, coping, relationships, pain, and senses [67]. Besides measuring health-related quality of life, the AQoL-6D is suitable for economic evaluations of health programs and has good psychometric properties [67]. It has also shown to be reliable, with a Cronbach’s alpha of .89 [68]. Because this is a relatively new instrument, we additionally will use the EuroQol (EQ-5D-5 L), the most widely used quality of life assessment, as a basis for cost-utility analyses [69]. The EQ-5D measures five health domains of importance to quality of life: mobility, self-care, usual activities, pain/discomfort, and anxiety/depression. The 5-level version includes five levels of response, which has been found to be more discriminative and to reduce ceiling effects compared to the 3-level version [70, 71].

#### Pain intensity and pain associated disability

Pain intensity and pain-related disability will be measured following the IMMPACT recommendations for core outcome measures for chronic pain clinical trials [72, 73]. Pain intensity will be measured by an 11-point numerical rating scale (0-10) of pain intensity as well as categorical classification of pain intensity (none, mild, moderate, severe). The Oswestry Disability Index (ODI) will be used to assess pain related disability. The ODI is a reliable and valid self-assessment questionnaire including 10 items [74].

#### Pain self-efficacy

Self-efficacy with regard to pain management will be assessed by using the Pain Self-Efficacy Questionnaire (PSEQ), which is a validated and reliable (internal consistency: α = 0.93) 10 item instrument that assesses self-efficacy expectations related to pain [75].

#### Work capacity

Work capacity will be assessed using the German version of the Subjective Prognostic Employment Scale (SPE), a validated 3-item self-report questionnaire with high internal consistency (Guttman scaling: rep = .99) [76].

#### Intervention satisfaction and adherence

Patient satisfaction with the intervention will be measured by using an adaptation of the Client Satisfaction Questionnaire (CSQ-8, German: ZUF-8), optimized for the assessment of client satisfaction with online interventions (CSWIQ-8) [77]. The CSQ-8 is a validated 8 item instrument with high internal consistency (α = 0.93) [78]. The adapted version, validated for the assessment of client satisfaction in web-based interventions, has been shown to have high internal consistency in a range of studies (α = 0.92-0.94) and is associated with treatment adherence and outcome [79–81]. The attrition rate (i.e. percentage of participants who no longer use the intervention assessed by their log in data) will give an estimate of the participants’ intervention adherence.

#### Side effects of psychotherapy

The German version of the Side Effects of Psychotherapy Inventory (INEP) [82] will be used to measure side-effects of psychotherapy. The INEP consists of 15 items assessing a range of common changes participants may have experienced due to the effects of the preventive intervention in their social and work environments.

#### Costs

To measure costs, the Dutch cost questionnaire: “Trimbos Institute and Institute of Medical Technology Questionnaire for Costs Associated with Psychiatric Illness” (TiC-P) [51], adapted for the German health care system, will be used [83]. The TiC-P is a widely used self-report questionnaire to measure health care consumption and productivity loss [84]. We further adapted the questionnaire for the population of chronic back pain patients. Participants will register all direct health service uptakes during the last 3 months, e.g. the number of general practice visits, sessions with psychiatrists, and hospital days. In addition, productivity-related costs will also be assessed. This includes the number of ‘work loss’ days (absenteeism), the number of ‘work cut-back’ days (presenteeism), and costs associated with for domestic tasks. Estimated development costs as well as opportunity costs will be included in the economic evaluation.

#### Covariates

As potentially effect-modifying covariates, demographic variables (gender, age, education), social support and medical variables (prior pain and depression treatments) will be assessed via self-report at baseline. Internet competencies will be assessed by the Internet Affinity Scale [85]. The translated version by Haase and colleagues [86] will be used. Back pain type, severity and chronicity will be extracted from medical records.

## Statistical analysis

### Clinical analyses

All analyzes will be performed according to the intention-to-treat (ITT) principle. Kaplan-Meier curves and Cox proportional hazard regression analysis will be used to determine differences in time to onset of MDD in weeks between both study conditions over a follow-up period of 12 months. The dependent variable will be time to onset of MDD and treatment condition will be the independent variable. The proportional-hazards assumption will be tested based on the scaled Schoenfeld residuals test. Survival analysis assumes that censoring (i.e., a participant is lost-to-follow-up or completes the follow-up period without experiencing a major depressive episode) is non-informative. Non-informative implies that the reasons why participants drop out of the trial are unrelated to the study condition (i.e., participants in one study arm should not be routinely censored). We will apply the following methods to deal with informative censoring, if necessary: (a) imputation techniques for missing data, (b) sensitivity analyses to illustrate best and worst case scenarios to test the robustness of the base case findings and (c) the use of the drop-out event as a study end point [87]. In addition, the number needed-to-treat (NNT) to prevent one additional event in the intervention group versus the control group will be calculated [88]. Covariates will first be checked whether they are associated with the primary outcome, if not left out of the final analysis.

Secondary outcomes will be analyzed using hierarchical linear modeling. To examine potentially moderating covariates, correlation of covariates and outcome parameter are analyzed using multiple regression models. In addition, per protocol analysis will be performed to investigate the influence of drop-outs on study results. Missing data will be imputed using multiple imputation. Potential confounding factors between source population and study population will be assessed, which enables us to evaluate the external validity of the sample. Finally, characteristics of dropped out participants at follow-up will be inspected and resulting socio-demographic differences between intervention and control group will be described. A significance level of *p* ≤ .05 will be set for all analyses.

### Economic evaluation

Baseline utilities and costs will be compared between both groups and if necessary, statistical techniques will be used to adjust for baseline differences [89]. In the cost-effectiveness analysis, the incremental cost-effectiveness ratio (ICER) will be presented as costs per depression-free year (DFY) gained. DFYs will be based on the number of depression-free weeks up to onset of a major depressive episode within the 12-month follow-up period. The ICER in the cost-utility analysis will be stated as costs per quality-adjusted life year (QALY) gained. Non-parametric bootstrapping (2500 times) will be applied to estimate the robustness of the ICERs and to quantify the uncertainty around the ratios. The bootstrapped replicates of the ICERs will be graphically represented in a cost-effectiveness plane, with effects along the horizontal axis and costs along the vertical axis.

In addition, a cost-effectiveness acceptability curve will be graphed to assess the probability that the intervention is more cost-effective relative to treatment as usual at varying willingness-to-pay (WTP) ceilings.

## Discussion

This study will be the first to investigate the effectiveness and cost-effectiveness of a psychological Internet- and mobile-based intervention for the prevention of depression in a chronic pain population. Due to its recruitment strategy from routine medical health care, the entire potential target group can be reached within a naturalistic setting. Results will have implications for researchers, health care providers and public health policy makers.

Conducting IMI-trials commonly involves possible limitations, which we try to overcome using the following measures. First, web-based interventions can have moderate to high drop-out rates [90–93], and drop-out rates can be expected to be even higher in preventive interventions due to the lower symptom burden of participants. We will approach this problem in different ways: a) by focusing on patients with current depressive symptoms b) by providing guidance via eCoaches, which has been shown to have an adherence-facilitating effect [35, 36], and c) by explicitly facilitating at risk participants’ motivation to use the intervention after discharge from orthopedic rehabilitation care. In a prior IMI with diabetes patients, participants completed an average of 78.3% of all sessions [44]. In a sample of subthreshold depressed patients, participants completed an average of 82.2% [31]. These results correspond to findings from a recent meta-analysis on adherence to internet-based CBT (ICBT) [94]. Van Ballegooijen and colleagues concluded that adherence to guided ICBT could be equal to adherence to face-to-face CBT. Participants do not necessarily have to complete all sessions to benefit from IMIs. They may also stop the treatment because they have recovered [95] or experienced improvement in symptoms, thereby reducing the likelihood of developing depression. These cases would represent a prevention success rather than a treatment drop-out [90].

A second limitation of most Internet- and mobile-based trials to date (including those mentioned above) are their highly selective online recruitment strategies. This may explain the promising results concerning drop-out rates, as participants already connected to the Internet comprised the intervention groups. As a down-side, however, those recruitment strategies lead to a lack of external validity [27, 96, 97]. In our study, we address this problem through the integration of the intervention into routine care. Thereby, the entire potential target group will be offered the opportunity to take part in the preventive intervention. The two different recruitment strategies will allow for analyses on different dissemination and implementation strategies of IMIs into routine healthcare. Thus, we can estimate what kind of patients, and to which extent, make use of the offer to take part in a preventive IMI within the whole group of chronic back pain patients.

Third, IMIs can have negative side effects [98–100]. For this reason, we followed the key recommendations of Rozental and colleagues [98]. We increased the flexibility of the treatment schedule by giving participants the possibility of delay at the beginning of each session, and increased flexibility of therapist contact for patients. Additionally, we prolonged treatment duration by adding two booster sessions after the main treatment modules. Furthermore, negative side effects of treatment will be assessed on a regular basis and reasons for drop-out from intervention will be assessed.

The specific strengths of this study are the following: a) Prevention studies are regularly methodologically limited because they lack a diagnosis at baseline and/or follow-up [6]. By carrying out the SCID prior to study start and at 12-months follow-up a high content validity can be ensured. b) With a target sample of 406 participants, the study will be optimally powered, overcoming the small scale trial limitations of most prior prevention studies [6, 20]. Following the ITT principle contributes to reducing overestimation of clinical effectiveness. c) The intervention is specifically tailored to the special needs of the target group of chronic back pain patients. This has been discussed as having an uptake and adherence facilitating effect [27]. We aim to further facilitate adherence through the integration of the intervention into patients’ routine healthcare, which enables clinicians to inform participants about the characteristics and effectiveness of IMIs. This may have a positive impact on their acceptance [101]. d) Using the internet as the medium for prevention might allow for scaling up of preventive interventions on a public mental health level. e) The direct implementation of the intervention into the health-care system increases external validity in contrast to prior RCTs [31, 102].

High prevalence rates underscore that the integration of depression prevention into curative care systems for the medically ill is one of the major emerging global health challenges. If this study - the first of its kind – shows to be effective, the intervention could be implemented into general (chronic) back pain and mental health treatment protocols as well as adapted to other chronically ill patient groups, thus helping to reduce the disease burden of depression for both affected persons and society. Thus, the results of this study will be of major public health relevance.

## Abbreviations

CBT: Cognitive behavioral therapy
CONSORT: Consolidated Standards of Reporting Trials
DFG: Deutsche Forschungsgemeinschaft (German Research Foundation)
DFY: Depression-free years
DRKS: Deutsches Register Klinischer Studien (German clinical studies trial register)
DSM: Diagnostic and Statistical Manual of Mental Disorders
DSMB: Data safety and monitoring board
ICBT: Internet-based cognitive behavioral therapy
ICC: Intra-class correlation
ICD: International classification of diseases
ICER: Incremental cost-effectiveness ratio
IMI: Internet- and mobile-based intervention
IMMPACT: Initiative on methods, measurement and pain assessment in clinical trials
IRR: Inter-rater reliability
ISO: International organization for standardization
ITT: Intention-to-treat
MDD: Major depressive disorder
NEN: Nederlandse Norm (Dutch Norm)
NNT: Number needed to be treated
PROD-BP: Prevention of depression in back pain patients
QUALY: Quality-adjusted life year
RCT: randomized controlled trial
SCID: Structured Clinical Interview for DSM
TAU: Treatment as usual
WHO: World health organization
WTP: Willingness-to-pay
